# Ammonia storm: unmasking a suspected rare proximal urea cycle disorder in adulthood

**DOI:** 10.1186/s12883-026-04996-1

**Published:** 2026-06-12

**Authors:** Vikram Vikhe, Md. Hassan Shaik, Priya Khaire, Rahul Patil, Ahanaa Chakraborty

**Affiliations:** General Medicine Department, Dr. D.Y. Patil Medical College, Hospital and Research Centre, Pune, India

**Keywords:** Hyperammonaemia, Urea cycle disorder, CPS-I deficiency, adult onset, Metabolic encephalopathy

## Abstract

**Background:**

Hyperammonaemia is a medical emergency which is mostly associated with liver dysfunction. However, in the absence of hepatic disease, rare inborn errors of metabolism such as urea cycle disorders (UCDs) must be considered. While UCDs typically present in the neonatal period, partial enzyme deficiencies may remain clinically silent until adulthood and present abruptly with life-threatening hyperammonaemia encephalopathy. Among these, late-onset carbamoyl phosphate synthetase I (CPS-I) deficiency is exceedingly rare and often underdiagnosed.

**Case presentation:**

We report a 32-year-old male who presented with acute onset altered sensorium progressing to coma over 12 h. The neurological decline was preceded by two to three episodes of vomiting on the day of admission and a recent history of a five-to-six-day febrile illness accompanied by gastrointestinal symptoms. On admission, the patient had a Glasgow Coma Scale score of 7/15 and required endotracheal intubation with ventilatory support. Routine laboratory investigations, including liver and renal function tests, were within normal limits. Neuroimaging studies (CT and MRI brain) showed no abnormalities. Plasma ammonia was markedly elevated at 397 µg/dL (Normal: <50 µg/dL), which rapidly escalated to 804 µg/dL within 24 h despite initial pharmacological intervention. Further metabolic evaluation revealed low plasma citrulline, elevated glutamine levels, and low urinary orotic acid, suggestive of a proximal urea cycle defect such as carbamoyl phosphate synthetase I (CPS-I) or N-acetylglutamate synthase (NAGS) deficiency. A significant family history of consanguinity and unexplained neonatal death, further supported a genetic etiology. The patient underwent urgent haemodialysis for ammonia clearance and was treated with protein restriction, dextrose-based intravenous fluids, and ammonia-lowering agents including sodium benzoate. His neurological status improved progressively, allowing successful extubation after three days. He was later discharged on oral L-citrulline supplementation and dietary protein restriction. At two-week follow-up, he was clinically stable with normalized plasma ammonia levels.

**Conclusion:**

This case highlights the importance of considering late-onset urea cycle disorders in adults presenting with unexplained hyperammonaemia encephalopathy. Early recognition and prompt aggressive management can be lifesaving and lead to complete neurological recovery.

## Introduction

Hyperammonemia is a serious metabolic condition characterized by elevated levels of ammonia in the blood and is mostly associated with acute or chronic liver dysfunction. Ammonia is a neurotoxin, and its accumulation can rapidly lead to cerebral edema, encephalopathy, seizures, coma, and death if not promptly recognized and treated [1]. In adult patients presenting with hyperammonemic encephalopathy and normal liver function tests, alternative etiologies must be actively explored, as delayed diagnosis can have catastrophic consequences. Urea cycle disorders (UCDs) are rare inborn errors of metabolism resulting from deficiencies of enzymes involved in ammonia detoxification through the urea cycle. These disorders classically present in the neonatal period with severe hyperammonemia; however, partial enzyme deficiencies may allow survival into adulthood with little or no symptoms until a metabolic stressor unmasks the condition [2, 3]. Such stressors include infections, fasting, increased protein intake, surgery, or certain medications, all of which can precipitate an acute hyperammonemic crisis [4].

Carbamoyl phosphate synthetase I (CPS-I) deficiency is one of the rarest urea cycle disorders and results from impaired conversion of ammonia to carbamoyl phosphate in hepatic mitochondria. Unlike ornithine transcarbamylase deficiency, CPS-I deficiency is not associated with elevated urinary orotic acid levels, a biochemical distinction that aids in diagnosis [5]. Late-onset CPS-I deficiency is particularly uncommon and poses a diagnostic challenge due to its nonspecific presentation and rarity in adult clinical practice [6]. Adult-onset UCDs are frequently misdiagnosed as toxic, infectious, psychiatric, or cerebrovascular conditions, leading to delays in appropriate management [7]. Neuroimaging is often normal in early stages, further complicating diagnosis [8]. Measurement of plasma ammonia levels, therefore, plays a crucial role in the early identification of metabolic encephalopathy and should be considered in all adults with unexplained altered sensorium [9].

Early recognition and aggressive management—including ammonia-lowering therapies, hemodialysis, and long-term dietary modifications—are essential to prevent irreversible neurological damage and improve outcomes [10]. We report a rare case of suspected late-onset partial CPS-I deficiency presenting as acute hyperammonemic encephalopathy in an adult, emphasizing the importance of maintaining a high index of suspicion for urea cycle disorders in similar clinical scenarios.

## Case presentation

A 32-year-old male was brought to the emergency department by family members with complaints of progressive alteration in sensorium over the preceding 12 h. The patient was reportedly well earlier in the day. At approximately 9:00 AM, he developed mild drowsiness but remained oriented and communicative. Over the next 5–6 h, his level of consciousness deteriorated progressively, with reduced verbal responsiveness, and by around 5:00 PM, he became unresponsive. This episode was associated with a single event of urinary incontinence. There was no history suggestive of seizure activity, head trauma, fall, alcohol intake, or exposure to toxins. The patient had three episodes of vomiting earlier the same day. The vomitus was non-projectile, non-bilious, non-blood stained, and contained food particles. A history of fever with loose stools was present three days prior to admission, lasting approximately five to six days, which resolved following treatment with oral antibiotics. There was no history of headache, visual disturbances, focal neurological deficits, or similar episodes in the past.

His past medical and surgical history was unremarkable, and he had no known comorbidities. Personal history revealed a mixed diet with normal appetite, sleep, bowel, and bladder habits. There was no history of substance abuse. Family history was significant for the unexplained death of a female sibling at four days of life. While this neonatal event suggests a potential metabolic etiology, no biochemical data were available to confirm the diagnosis in the sibling. The patient’s younger brother was healthy. There was a history of consanguineous marriage between his parents. On examination at presentation, the patient was afebrile and hemodynamically stable. Neurological assessment revealed a Glasgow Coma Scale (GCS) score of E2 V2 M3 (7/15). Pupils were equal and reactive to light, and there were no signs of meningeal irritation or focal neurological deficits. Plantar responses were flexor bilaterally. There were no clinical stigmata of chronic liver disease such as jaundice, spider nevi, palmar erythema, or ascites. Respiratory, cardiovascular, and abdominal examinations were unremarkable. In view of the low GCS and risk of airway compromise, the patient was electively intubated and placed on mechanical ventilatory support.

Initial laboratory investigations, including complete blood count, renal function tests, liver function tests, serum electrolytes, calcium, magnesium, phosphorus, urine routine microscopy, hepatitis B surface antigen, and anti-hepatitis C virus antibodies, were within normal limits, effectively excluding hepatic, renal, and infective etiologies. A urine toxicology panel was also sent which came out as normal. Plasma ammonia levels sent at admission came markedly elevated at 397 µg/dL. Non-contrast computed tomography of the brain was normal. Magnetic resonance imaging of the brain with contrast showed no parenchymal or meningeal abnormalities. Initial supportive management consisted of 10% dextrose-containing intravenous fluids to maintain an anabolic state and a loading dose of intravenous sodium benzoate 5.5 g/m^2^ Body Surface Area(BSA) over 90–120 min, followed by maintenance dose of 5.5 g/m^2^ BSA IV infusion over 24 h. Despite these measures, plasma ammonia levels rose to 804 µg/dL after 24 h. Given the rapidly rising plasma ammonia levels, an urgent nephrology consultation was obtained, following which the patient underwent one 4-hour session of Intermittent hemodialysis, successfully reducing ammonia levels to 110 µg/dL. The patient showed significant neurological improvement and was successfully extubated after three days. Chest radiograph (posteroanterior view) was unremarkable. Ultrasonography of the abdomen and pelvis revealed mild free fluid in the pelvis, with no other abnormalities. Electroencephalography was normal.

**Fig. 1:**
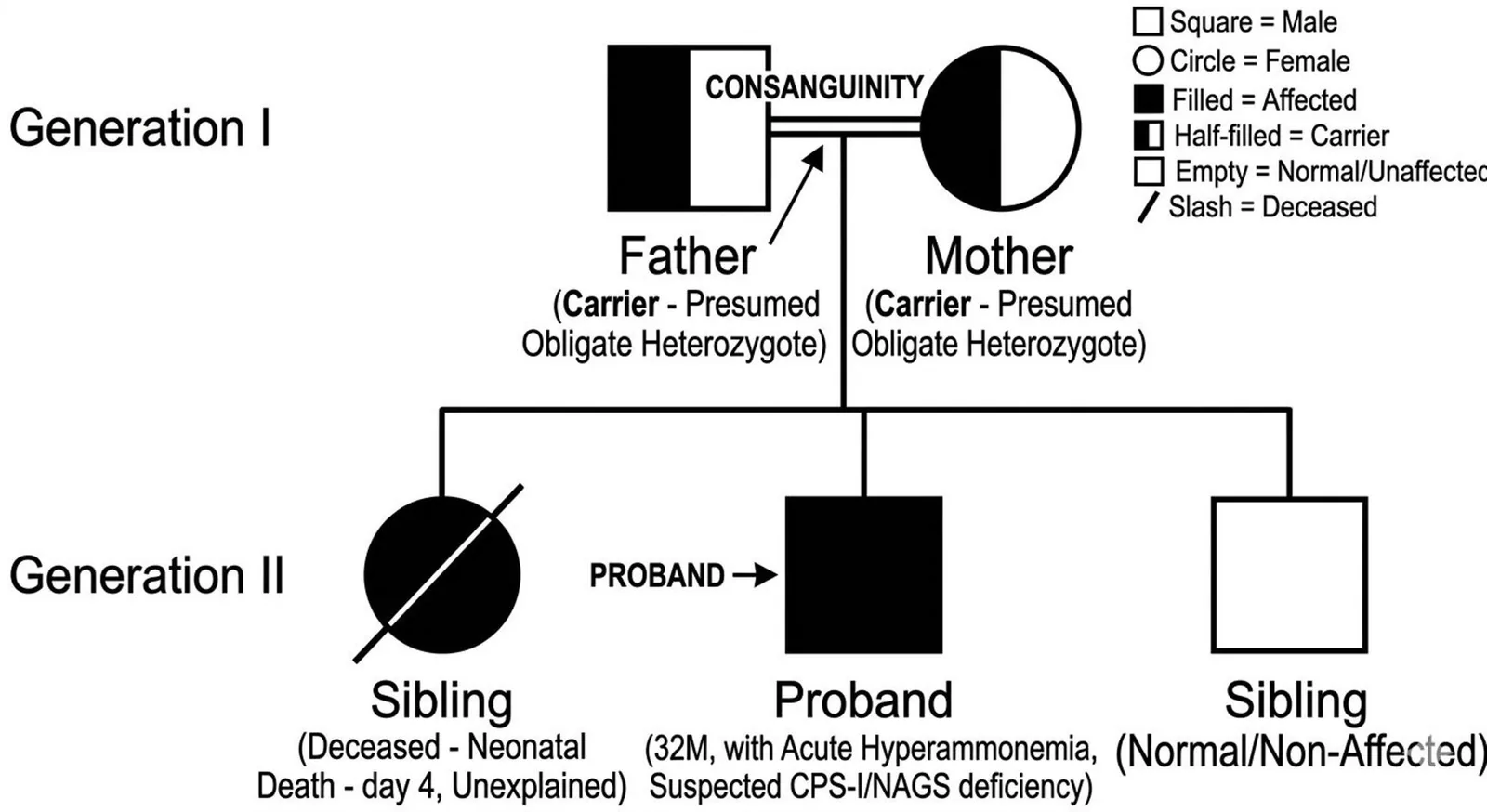
Pedigree chart of the family. The chart illustrates parental consanguinity and the suspected neonatal death of a sibling. In the absence of biochemical data for the deceased neonate, the familial link to the proband’s metabolic disorder remains a clinical suspicion rather than a confirmed diagnosis. Parents are represented as presumed obligate heterozygotes

Given the severe hyperammonemia in the absence of liver dysfunction, further metabolic investigations were pursued. Plasma and urine quantitative amino acid analysis demonstrated low plasma citrulline levels with elevated glutamine levels. Urinary orotic acid levels were low, findings consistent with proximal urea cycle defect, specifically carbamoyl phosphate synthetase I(CPS-I)deficiency and N-acetylglutamate synthase (NAGS) deficiency. CPS-I deficiency was considered the primary clinical suspicion given its more frequent association with this specific metabolic presentation in adults and the patient’s significant family history. Although genetic confirmation testing to distinguish between the two could not be done due to financial constraints, the clinical course and response to ammonia scavenging therapy were consistent with a late-onset partial CPS-I defect. The absence of metabolic acidosis and ketonuria, alongside normal urine organic acid analysis, helped exclude organic acidurias. Management was done with protein-restricted diet, dextrose-containing intravenous fluids to prevent catabolism, and intravenous sodium benzoate. He was discharged on oral L-citrulline supplementation and dietary protein restriction. At two-week follow-up, the patient remained asymptomatic. Repeat biochemical investigations showed a normalised plasma ammonia of 32 µg/dL. Furthermore, follow-up plasma amino acid analysis revealed a marked decline in glutamine levels(from an initial peak of > 1000 µmol/L to 580 µmol/L; Normal:350–750 µmol/L). (Fig. 1).

## Discussion

Hyperammonemia in adults is most frequently associated with hepatic dysfunction; however, when liver function tests are normal, alternative, and often rare etiologies must be considered. Urea cycle disorders (UCDs) represent an important but underrecognized cause of non-hepatic hyperammonemia in adults and are associated with significant morbidity and mortality if not promptly diagnosed and treated [1, 2]. This case illustrates the diagnostic challenge of adult-onset hyperammonemia in the absence of hepatic disease. The combination of low plasma citrulline and low urinary orotic acid points toward a defect in the proximal steps of the urea cycle. While we suspected late-onset CPS-I deficiency, NAGS deficiency remains a critical differential as both present with identical biochemical profiles.

The phenotypic disparity observed in this family-characterised by a neonatal fatality in one sibling and a late-onset adult presentation in the proband-presents a complex clinical picture common to partial enzymatic defects. In such instances, residual enzyme activity may suffice for normal metabolic function until an acute catabolic event, such as the preceding febrile illness and gastrointestinal distress reported here, creates a surge in nitrogeneous waste that overwhelms the restricted capacity of the urea cycle. However, a definitive familial link remains speculative. While the neonatal death is highly suggestive of a more severe manifestation of an identical metabolic disorder, the absence of objective data or neonatal ammonia levels for the deceased sibling rules out a conclusive diagnosis. The sibling’s death may represent a complete enzyme deficiency or an unrelated neonatal pathology. Consequently, the family history serves as a significant clinical indicator of a potential genetic etiology but must be interpreted with the necessary scientific caution regarding its diagnostic certainty.

The present case highlights a suspected rare instance of late-onset carbamoyl phosphate synthetase I(CPS-I) deficiency presenting as acute hyperammonemic encephalopathy in adulthood. UCDs are inherited metabolic disorders caused by deficiencies of enzymes involved in ammonia detoxification. CPS-I deficiency affects the first and rate-limiting step of the urea cycle and is typically associated with severe neonatal hyperammonemia. However, partial enzyme deficiencies may allow individuals to remain asymptomatic until a metabolic stressor precipitates an acute crisis [3]. In this patient, the antecedent febrile illness acted as the precipitating catabolic trigger. Fever and gastrointestinal distress increase metabolic demands, leading to the breakdown of endogenous proteins. In the setting of a partial CPS-I deficiency, this surge in nitrogenous waste overwhelmed the restricted capacity of the urea cycle, culminating in a life threatening ‘ammonia storm’.

Clinical manifestations of hyperammonemia are largely neurological and nonspecific, ranging from mild confusion to coma and cerebral oedema [4]. Neuroimaging findings may be normal in the early stages, as observed in this patient, which can delay diagnosis [5]. Therefore, measurement of plasma ammonia levels is critical and should be included in the evaluation of any adult presenting with unexplained altered sensorium [6]. The markedly elevated ammonia levels in the absence of liver disease were key diagnostic clues in this case. Biochemical profiling plays a pivotal role in differentiating among UCD subtypes. The combination of low plasma citrulline, elevated glutamine levels, and low urinary orotic acid strongly suggested CPS-I deficiency and helped distinguish it from ornithine transcarbamylase deficiency, the most common UCD presenting in adults [7, 8]. Although genetic confirmation could not be obtained due to financial constraints, the biochemical pattern and clinical presentation were highly supportive of the diagnosis.

While inborn errors of metabolism like CPS-I deficiency are critical genetic considerations, several acquired conditions can precipitate unexplained adult-onset hyperammonemia in the absence of primary liver disease. Portosystemic shunts, whether congenital or acquired, may allow ammonia rich portal blood to bypass hepatic metabolism even in the absence of cirrhosis. Additionally, several medications are known triggers; Valproic acid can interfere with urea cycle by inhibiting CPS- I enzyme activity and also disrupts mitochondrial function. Also certain chemotherapeutic agents(e.g. 5-fluorouracil) can acutely elevate ammonia levels. Infections with urea-splitting organisms(such as Proteus or Klebsiella) can also cause significant systemic ammonia absorption. Furthermore, states of extreme catabolism, including multiple myeloma, severe malnutrition, or high protein total parenteral nutrition, can overwhelm the urea cycle’s capacity even in previously asymptomatic individuals. In this case, the lack of offending medications and the presence of significant family history pointed toward a genetic etiology rather than an acquired one.

Differentiating between proximal UCD subtypes is clinically vital for targeted management. Specifically, NAGS deficiency shows a dramatic clinical and biochemical response to carglumic acid, a structural analogue of N-acetylglutamate that restores CPS- I activity. Conversely, patients with primary CPS- I deficiency typically do not respond to this agent. While our primary clinical suspicion remained late-onset CPS- I deficiency given the patient’s presentation, a therapeutic trial or diagnostic consideration of carglumic acid remains an essential step in the management of suspected proximal defects to definitively rule out NAGS deficiency. Management of acute hyperammonemia requires rapid reduction of ammonia levels to prevent irreversible neurological damage. Hemodialysis remains the most effective modality for rapid ammonia clearance in severe cases [9]. Early recognition and the prompt use of hemodialysis for levels exceeding 500 µg/dL are essential to prevent irreversible cerebral edema Adjunctive therapies, including ammonia scavengers such as sodium benzoate, caloric supplementation to prevent catabolism, and dietary protein restriction, are essential components of both acute and long-term management [10]. Early intervention in this case resulted in complete neurological recovery. This case underscores the importance of maintaining a high index of suspicion for late-onset UCDs in adults with unexplained hyperammonemic encephalopathy.

## Conclusion

This case highlights a rare presentation of suspected late-onset carbamoyl phosphate synthetase I deficiency manifesting as acute hyperammonemic encephalopathy in adulthood. In patients presenting with unexplained altered sensorium and markedly elevated ammonia levels in the absence of liver disease, urea cycle disorders should be strongly considered. Early recognition, prompt metabolic evaluation, and aggressive ammonia-lowering interventions, including hemodialysis, are critical to prevent irreversible neurological damage. Long-term dietary modification and appropriate supplementation play a key role in preventing recurrence. Awareness of this rare but treatable condition can significantly improve patient outcomes and reduce mortality.

## Data Availability

No datasets were generated or analysed during the current study.
